# Betatrophin Acts as a Diagnostic Biomarker in Type 2 Diabetes Mellitus and Is Negatively Associated with HDL-Cholesterol

**DOI:** 10.1155/2015/479157

**Published:** 2015-12-24

**Authors:** Min Yi, Rong-ping Chen, Rui Yang, Xian-feng Guo, Jia-chun Zhang, Hong Chen

**Affiliations:** Department of Endocrinology, Zhujiang Hospital, Southern Medical University, Guangzhou 510280, China

## Abstract

*Objective*. By assessing its circulating concentrations in type 2 diabetes mellitus (T2DM) patients, we aimed to explore the associations of betatrophin with various metabolic parameters and evaluate its diagnostic value in T2DM.* Methods*. A total of 58 non-diabetes-mellitus (NDM) subjects and 73 age- and sex-matched newly diagnosed T2DM patients were enrolled. Correlation analyses between circulating betatrophin levels and multiple metabolic parameters were performed. Receiver operating characteristic (ROC) curve analysis was used to assess the diagnostic value of betatrophin concentration in T2DM.* Results*. Circulating betatrophin levels were approximately 1.8 times higher in T2DM patients than in NDM individuals (median 747.12 versus 407.41 pg/mL, *P* < 0.001). Correlation analysis showed that betatrophin was negatively associated with high-density lipoprotein cholesterol (HDL-C) levels in all subjects. ROC curve analysis identified betatrophin as a potent diagnostic biomarker for T2DM. The optimal cut-off point of betatrophin concentration for predicting T2DM was 501.23 pg/mL.* Conclusions*. Serum betatrophin levels were markedly increased in newly diagnosed T2DM patients and further elevated in obese T2DM subjects. Betatrophin was negatively correlated with HDL-C levels. Our findings indicate that betatrophin could be a potent diagnostic biomarker for T2DM.

## 1. Introduction

Diabetes mellitus prevalence is increasing at alarming rates, and this ailment has become a major public health problem worldwide. According to the International Diabetes Federation (IDF), 360 million individuals suffered from diabetes in 2011, a number expected to rise to 522 million with a prevalence of 7.7% in 2030 [1]. Type 2 diabetes, characterized by insulin resistance and pancreatic *β* cell function defect, makes up about 90% of all cases [2]. Replenishing insulin-producing pancreatic *β* cell mass and alleviating insulin resistance are considered the ideal ways for diabetes care. Targeting the pathophysiological defects that characterize the onset of diabetes can achieve a durable glucose control and benefit to essential components in disease pathogenesis.

Betatrophin, a newly characterized circulatory hormone secreted by liver and adipose tissues, is believed to promote *β* cell proliferation, therefore attracting increasing attention. After injection of the insulin receptor antagonist S961 into mice, Melton and colleagues discovered a secreted protein of 198 amino acids that specifically induces dramatic and specific pancreatic *β* cell proliferation, improving glucose tolerance [3]. The notion that betatrophin may interfere with the compensatory response to insulin resistance has raised hope for new diabetes therapeutic in humans. However, other studies found that mouse betatrophin has no effect on human beta cell proliferation and differentiation [4, 5]. In addition, overexpression and silencing of the betatrophin gene in mice do not support a role for this hormone in controlling beta cell growth but point to a clear function in regulating plasma lipid profiles [6]. Meanwhile, opinions regarding the associations of betatrophin with T2DM and obesity in humans are also discrepant. Some studies suggested that circulating betatrophin levels are elevated in type 2 diabetes and obesity [7–10], correlating with lipid profiles, while others reported that betatrophin is associated only with lipid metabolism and has nothing to do with glucose homeostasis [11, 12]. Is it possible that betatrophin is involved in T2DM development and lipid metabolism? Whether betatrophin is a potential target for diabetes and dyslipidemia medications or solely a diagnostic biomarker remains poorly understood. Therefore, we assessed serum betatrophin concentrations in Chinese population. We hypothesized that betatrophin levels might be increased in obese individuals with T2DM and constitute a potential diagnostic biomarker for T2DM.

## 2. Materials and Methods

### 2.1. Study Population

From 2013 to 2014, a total of 131 Chinese subjects (69 males and 62 females) were recruited at the Diabetes Clinics and Medical Examination Center of Zhujiang Hospital. Eligible patients were males and females over 18 years, including 58 non-diabetes-mellitus subjects (NDM: 18 lean, 22 overweight, and 18 obese individuals) and 73 age- and sex-matched patients with T2DM (22 lean, 29 overweight, and 22 obese individuals). T2DM was diagnosed according to the World Health Organization (WHO) diagnostic criteria for diabetes [13]. The exclusion criteria are as follows: (1) subjects being treated with oral hypoglycemic agents and those with macrovascular complications; (2) subjects taking any medications known to affect glucose tolerance within one month; (3) individuals with type 1 diabetes or gestational diabetes; (4) subjects with viral hepatitis, cancer, severe psychiatric disturbances, hepatic failure, chronic renal failure on hemodialysis, congestive heart failure, or other known major diseases. All subjects enrolled provided written informed consent. The study protocol was in agreement with the guidelines of the Human Research Ethics Committees of Zhujiang Hospital and performed in accordance with the ethical principles of the Declaration of Helsinki.

### 2.2. Anthropometric and Biochemical Measurements

All subjects underwent comprehensive anthropometric measurements, including height, weight, and waist and hip circumferences, whereby body mass index (BMI) and waist-to-hip ratio (WHR) were calculated. Weights were measured in light clothing without shoes. Heights were obtained with a portable, rigid measuring rod. BMI was derived as body weight divided by body height squared. Waist circumference was measured at the midpoint between the lowest rib margin and the iliac crest in a standing position. Hip circumference was measured at the widest point.

Blood samples were collected after 8 hours of fasting without taking any medications, for the assessment of fasting plasma glucose, insulin level, C peptide, total cholesterol (TC), triglycerides (TG), low-density lipoprotein cholesterol (LDL-C), high-density lipoprotein cholesterol (HDL-C), uric acid, aspartate aminotransferase (AST), alanine aminotransferase (ALT), and betatrophin concentrations. Serum betatrophin levels were determined with a commercially available human ELISA kit (Wuhan Eiaab Science, Wuhan, China; Catalogue number E11644h) according to the manufacturer's instructions. ELISA was performed in duplicate, and samples with coefficient of variation (CV) values exceeding 5% were excluded. A standard curve was constructed by plotting mean OD_450_ for each standard against its concentration, generating a best fit curve through the graph points. Fasting plasma glucose amounts were measured by the glucose oxidase method; fasting insulin and C peptide levels were measured by enzyme-amplified chemiluminescence assays. Serum TC, triglycerides, LDL-C, and HDL-C were assessed by enzymatic methods; ALT and AST were quantitated by kinetic methods (Beckman Coulter Inc., Brea, CA); serum uric acid levels were evaluated by the uricase method. Obesity was defined as BMI ≥ 30 kg/m^2^ and overweight was defined as a BMI between 25 and 30 kg/m^2^. Insulin resistance was estimated by homeostasis model assessment of insulin resistance (HOMA-IR) and Quantitative Insulin Sensitivity Check Index (QUICKI) [14]. Pancreatic *β* cell function was assessed by homeostasis model assessment of *β* cell function (HOMA-%*β*) [15]. HOMA-IR and HOMA-%*β* were derived using the following equations: HOMA-IR = insulin [lU/mL] *∗* glucose [mmol/L]/22.5; HOMA-%*β* = 20 *∗* insulin [lU/mL]/(glucose [mmol/L] − 3.5).

### 2.3. Statistical Analysis

All statistical analyses were performed using SPSS version 20.0 (SPSS, Inc., Chicago, IL, USA). Normally distributed and continuous variables were presented as mean ± standard deviation (SD) and nonnormally distributed variables as median and quartiles (25% and 75%). Comparisons between groups were assessed by independent-samples *t*-test; alternatively, analysis of variance (ANOVA) followed by LSD tests was conducted as appropriate. Correlations between variables were assessed using Pearson correlation analysis, controlling for covariates. Two-tailed *P* < 0.05 was considered statistically significant. Performance of betatrophin concentration in detecting T2DM was evaluated using receiver operating characteristic (ROC) curve analysis. Based on ROC analysis, the best cut-off value for betatrophin concentration was determined from the highest Youden index, which is defined as sensitivity + specificity − 1.

## 3. Results

### 3.1. Circulating Betatrophin Levels Are Significantly Increased in T2DM and Obese Subjects

**Figure 1:**
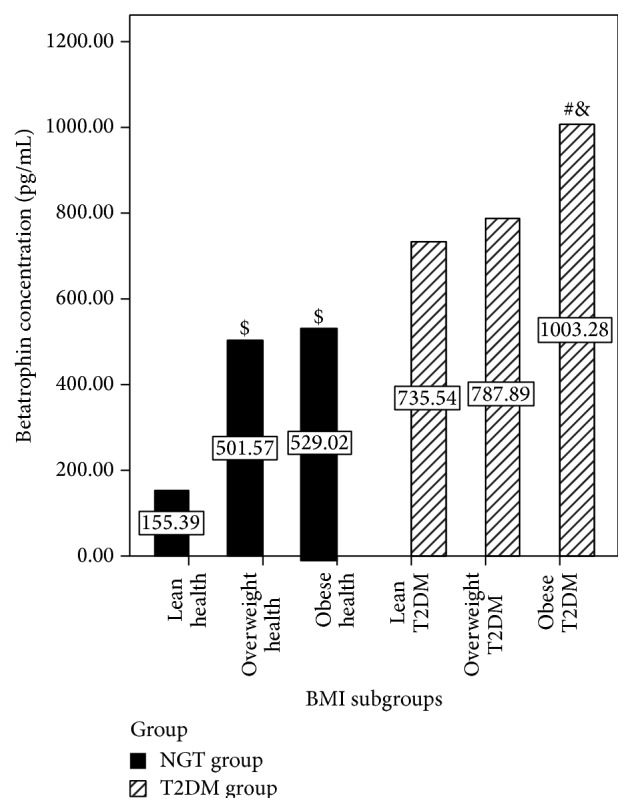
Serum betatrophin concentrations in each subgroup. Serum betatrophin concentration in NDM group: lean health (*n* = 18), overweight health (*n* = 22), and obese health (*n* = 18). Type 2 diabetes group: lean T2DM (*n* = 22), overweight T2DM (*n* = 29), and obese T2DM (*n* = 22). Data are mean ± SEM; ^$^ *P* < 0.05 versus lean health; ^#^ *P* < 0.05 versus lean T2DM; ^&^ *P* < 0.05 versus overweight T2DM.

A total of 58 NDM subjects and 73 age- and sex-matched T2DM patients were assessed in this study. Baseline characteristics of all participants are shown in Table 1. No significant differences were found in age, gender, BMI, WHR, TG, LDL-C, ALT, and AST levels between the two patient groups. Interestingly, serum betatrophin concentrations were approximately 1.8 times higher in T2DM patients than in NDM individuals (median 747.12 versus 407.41 pg/mL, *P* < 0.001). When stratified by BMI, betatrophin levels in obese T2DM subjects were almost 6.5 times higher than values obtained for healthy NDM subjects (1003.28 versus 155.29 pg/mL, *P* < 0.001, Figure 1).

### 3.2. Betatrophin Is Negatively Associated with HDL-C Levels in All Subjects

**Figure 2:**
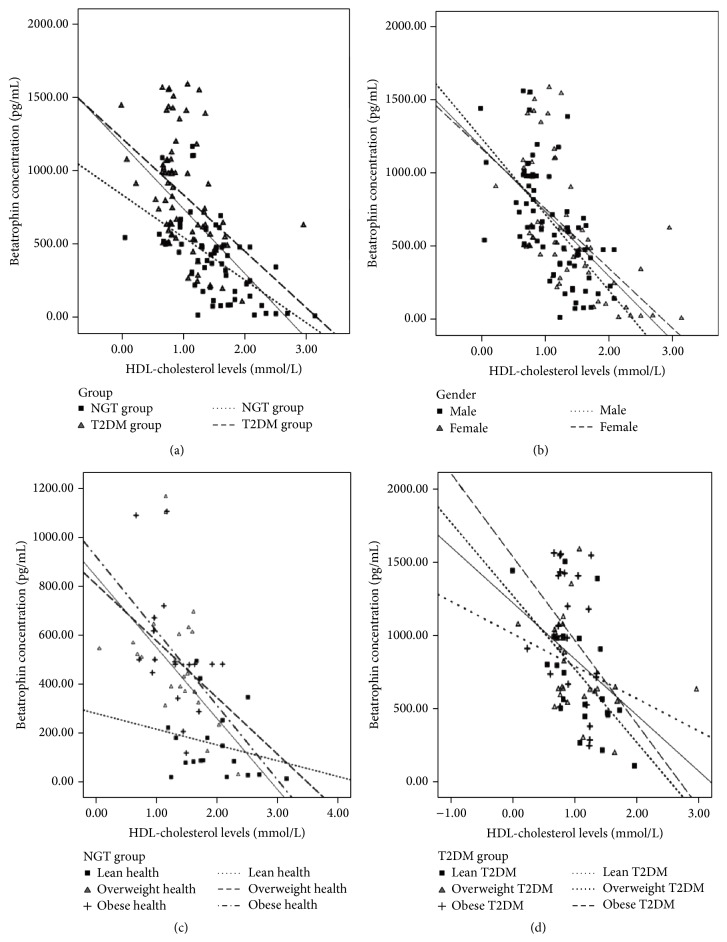
Correlations of serum betatrophin levels with HDL-cholesterol levels in each subgroup. (a) Correlations of serum betatrophin levels with HDL-cholesterol in NDM and T2DM groups. (b) Correlations of serum betatrophin levels with HDL-cholesterol in males and females. (c) Correlations of serum betatrophin levels with HDL-cholesterol in NDM group when stratified by BMI. (d) Correlations of serum betatrophin levels with HDL-cholesterol in T2DM group when stratified by BMI.

Next, we assessed the associations of circulating betatrophin levels with various metabolic parameters. In the T2DM group, betatrophin amounts were correlated only with age and HDL-cholesterol levels; meanwhile, serum betatrophin concentrations were associated with multiple metabolic parameters, including fasting glucose, WHR, insulin, C peptide, HOMA-IR, QUICK index, and lipid profiles (TG and HDL-C) in the NDM group (Table 2). When stratified by BMI, betatrophin is negatively associated with HDL-C levels in all subgroups (Figure 2).

### 3.3. Performance of Betatrophin Concentration in Detecting T2DM

**Figure 3:**
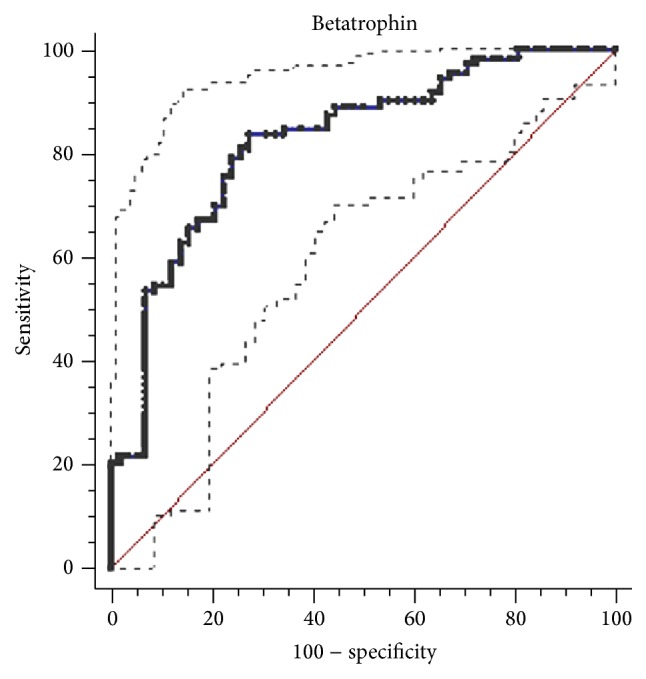
Receiver operating characteristics (ROC) curves showing the performance of betatrophin concentration in detecting type 2 diabetes mellitus. The receiver operating characteristic curve analysis. The optimal cut-off point was 501.23 pg/mL. The area under the ROC curve (area between the solid line and *x*-axis) was 0.824 (95% CI, area between the dashed lines and *x*-axis, 0.748–0.885, *P* < 0.001). The sensitivity and specificity are 83.56% and 72.41%, respectively.

The ROC curve shown in Figure 3 depicts the diagnostic accuracy of betatrophin level for T2DM. The optimal cut-off point (betatrophin concentration) to predict T2DM was 501.23 pg/mL. Using this cut-off value, diagnostic efficiency for T2DM reached the highest value: the area under the ROC curve was 0.824 (95% CI 0.748–0.885, *P* < 0.001), with sensitivity and specificity of 83.56% and 72.41%, respectively.

## 4. Discussion

Betatrophin is believed to promote pancreatic beta cell proliferation and to improve metabolic control by increasing beta cell division rate [3, 16] in insulin resistant mice. In humans, the associations of serum betatrophin levels with diabetes, obesity, and lipid profiles remain controversial [7, 9–12, 17–19]. We found that circulating betatrophin concentrations were significantly increased in T2DM and obese patients. Interestingly, for the first time, this study demonstrated that betatrophin was negatively correlated with HDL-C levels in both NDM and T2DM groups. Using a ROC curve, we found that circulation betatrophin concentration could be a diagnostic biomarker for T2DM, with optimal cut-off point of 501.23 pg/mL.

As shown above, serum betatrophin levels were approximately 1.8 times higher in T2DM patients than in NDM individuals; in addition, they were almost 6.5 times higher in obese T2DM subjects compared with values obtained for healthy NDM subjects. These findings corroborate several studies [7, 17, 20, 21] but contradict data reported by Gómez-Ambrosi et al. [11] showing significantly decreased circulating betatrophin in obese individuals, with further drop in IGT and T2DM participants. Several possible reasons may explain these conflicting results: firstly, different study populations and ethnicities were assessed. Increasing evidence shows that betatrophin mRNA is originally expressed in liver and white and brown adipose tissues [22, 23]; therefore, adipose distribution differences between the Europeans and Asians may affect betatrophin concentrations. In addition, T2DM patients assessed in the latter study were taking hypoglycemic medications. Since the effects of hypoglycemic agents on serum betatrophin levels are unclear, it would be challenging to distinguish the potential confounding effects. Lastly, betatrophin concentrations were determined by ELISA kits manufactured by different companies in both studies. Betatrophin proteolytic regulation leads to different circulating protein levels. Betatrophin ELISA kits may detect either the N- or C-terminus of the protein [24], resulting in differential or even conflicting data.

Our research revealed that betatrophin concentrations were negatively associated with HDL-C levels in both NDM and T2DM groups for the first time. So far, multiple animal-based studies have proposed that betatrophin level is closely related to altered blood lipid metabolism [22, 23, 25, 26]. Mice lacking the betatrophin gene (Gm6484) display lower serum triacylglycerol (TAG) levels, associated with reduced amounts of very low-density lipoprotein (VLDL) as well as elevated lipoprotein lipase (LPL) activity, whereas adenovirus-mediated betatrophin overexpression can elevate circulating triacylglycerol levels [23, 25]. Betatrophin may affect blood lipid profiles by regulating hepatic VLDL secretion and altering LPL activity. Betatrophin induces triglyceride elevation through reduced triglyceride clearance by inhibiting LPL activity. In addition, betatrophin may also act via a functional interaction with ANGPTL3 [23, 27], a known lipid regulator in mice and humans, which regulates HDL-C levels by inhibiting endothelial lipase [28]. Plasma TAG levels are unchanged in mice expressing ANGPTL3 alone, whereas coexpression with betatrophin results in hypertriglyceridemia despite a reduction in circulating ANGPTL3, suggesting betatrophin may regulate lipid metabolism by activating ANGPTL3 [25]. In accordance with animal studies, we found a significant association between serum betatrophin and triglyceride levels in this clinical trial. According to genome-wide association studies, betatrophin sequence variations are related to blood lipid levels in humans [23]. A betatrophin transcript variant and its expression levels are associated with clinical or pathological symptoms. For instance, single-nucleotide polymorphism in the betatrophin gene substituting tryptophan for arginine at residue 59 is reportedly associated with reduced HDL-C and LDL-C in African American and Hispanic participants [5]. Notably, we found that betatrophin was negatively correlated with HDL-C levels in all subjects, including the NDM and T2DM groups. When stratified by BMI, these trends still exist in all subgroups.

Another novel finding of this study is that betatrophin could be used as a diagnostic biomarker for T2DM. By performing ROC curve analysis, we found that circulation betatrophin concentration is a potent diagnostic biomarker for T2DM, with an optimal cut-off point of 501.23 pg/mL (AUROC = 0.824, 95% CI, 0.748–0.885, and *P* < 0.001). The notion that betatrophin could induce pancreatic beta cell proliferation has attracted attention at the very beginning [3, 29, 30]; however, further studies in betatrophin/Angptl8 knockout mice do not support a role for betatrophin in controlling beta cell growth [4, 6, 27, 31]. Meanwhile, new data showing that betatrophin levels are elevated in diabetes but not correlated with glucose homeostasis have greatly questioned the ability of betatrophin to increase beta cell replication in humans [18]. Betatrophin is much more likely to be a diagnostic biomarker rather than a potential therapeutic target for type 2 diabetes [5]. Our research, for the first time, assessed the diagnostic value of betatrophin in type 2 diabetes. Diabetes autoantibodies constitute an important indicator to distinguish type 1 from type 2 diabetes. The current testing method is complicated and more expensive. If betatrophin can be a reliable diagnostic biomarker and classification index for diabetes, it will allow great savings in medical work and expenses. However, our studies did not enroll type 1 diabetes patients; thus, future studies are needed to test this hypothesis.

Other limitations of this study should be taken into account. Firstly, the sample size was relatively small in this single center study, which may affect the statistical power. Secondly, the present research was a cross-sectional study, which could not address the cause-effect relationship between serum betatrophin and T2DM as well as obesity. Lastly, our cross-sectional trial only estimated serum betatrophin levels at a single point, which cannot reflect betatrophin levels over time.

In conclusion, although not definitive our data confirmed previous findings: increased serum betatrophin levels in T2DM and obese subjects. In addition, we observed for the first time a negative correlation between serum betatrophin levels and HDL-cholesterol amounts in all subjects. Our findings indicate for the first time that circulation betatrophin concentrations could be a diagnostic biomarker for T2DM. These intriguing results warrant further researches; for example, prospective studies should be carried out to clarify the detailed mechanism and association of betatrophin with T2DM and lipid profiles.

## Supplementary Material

Raw data of all the participants. Note: gender: 1 represents male; 2 represents female; groups: 1 represents non diabetes subject; 2 represents T2DM subjects; BMI subgroups: 1 represents lean subject without daibetes; 2 represents overweight subject without daibetes; 3 represents obese subjects without daibetes; 4 represents lean subject with T2DM; 5 represents overweight subject with T2DM; 6 represents obese subjects with T2DM.

## Figures and Tables

**Table 1:** General characteristics of normal glucose participants and newly diagnosed T2DM patients.

Variables	NDM	T2DM	*P* value
Gender (male/*N*)	31/58 (53.4%)	38/73 (52.1%)	0.874
Age (years)	39.43 ± 12.08	41.03 ± 9.74	0.404
BMI (kg/m^2^)	26.80 ± 4.26	27.66 ± 3.39	0.204
WHR	0.91 ± 0.07	0.93 ± 0.06	0.100
Betatrophin (pg/mL)	407.41 (172.76–528.32)	747.12 (544.26–1074.96)	<0.001^*∗*^
Fasting glucose (mM)	5.51 ± 0.63	12.11 ± 3.89	<0.001^*∗*^
Fast insulin (mIU/L)	10.42 ± 5.83	15.10 ± 8.18	<0.001^*∗*^
C peptide (*μ*g/L)	1.65 ± 0.76	2.58 ± 2.97	0.021^*∗*^
HOMA-IR	2.88 ± 1.98	7.90 ± 3.89	<0.001^*∗*^
HOMA-%*β*	92.45 ± 46.83	44.77 ± 37.37	<0.001^*∗*^
QUICKI	0.34 ± 0.04	0.29 ± 0.02	<0.001^*∗*^
TC (mM)	4.89 ± 1.23	5.47 ± 1.15	0.007^*∗*^
TG (mM)	1.29 (0.71–2.06)	1.66 (1.09–2.35)	0.213
LDL-C (mM)	3.32 ± 0.92	3.42 ± 0.95	0.559
HDL-C (mM)	1.50 ± 0.47	1.01 ± 0.37	<0.001^*∗*^
Uric acid (*μ*mol/L)	378.32 ± 110.26	335.40 ± 93.81	0.018^*∗*^
ALT (IU/L)	21 (14–37.8)	21 (14.5–31)	0.308
AST (IU/L)	24.28 ± 9.18	20.99 ± 12.48	0.096

BMI: body mass index; WHR: waist-to-hip ratio; FINS: fasting blood insulin; FBG: fasting blood glucose; HOMA-IR: homeostasis model assessment of insulin resistance; HOMA-%*β*: homeostasis model assessment of *β* cell function; TC: total cholesterol; TG: triglyceride; HDL-C: high-density lipoprotein cholesterol; LDL-C: low-density lipoprotein cholesterol; QUICKI: Quantitative Insulin Sensitivity Check Index; ALT: alanine aminotransferase; AST: aspartate aminotransferase.

^*∗*^ *P* < 0.05.

**Table 2:** Correlations between betatrophin levels and various parameters of the study subjects.

Variables	Overall	NDM	T2DM
Age (years)	*r* = −0.213; *P* = 0.813	*r* = −0.249; *P* = 0.059	*r* = 0.273; *P* = 0.020^*∗*^
BMI (kg/m^2^)	*r* = 0.325; *P* < 0.001^*∗*^	*r* = 0.240; *P* = 0.070	*r* = 0.162; *P* = 0.170
WHR	*r* = 0.199; *P* = 0.023	*r* = 0.403; *P* = 0.002^*∗*^	*r* = −0.003; *P* = 0.977
Fasting glucose (mM)	*r* = −0.465; *P* < 0.001^*∗*^	*r* = 0.474; *P* < 0.001^*∗*^	*r* = 0.141; *P* = 0.233
Fast insulin (mIU/L)	*r* = 0.351; *P* < 0.001^*∗*^	*r* = 0.448; *P* < 0.001^*∗*^	*r* = 0.067; *P* = 0.575
C peptide (*μ*g/L)	*r* = 0.184; *P* < 0.001^*∗*^	*r* = 0.354; *P* = 0.006^*∗*^	*r* = 0.057; *P* = 0.631
HOMA-IR	*r* = 0.461; *P* < 0.001^*∗*^	*r* = 0.432; *P* = 0.001^*∗*^	*r* = 0.129; *P* = 0.278
HOMA-%*β*	*r* = −0.249; *P* = 0.004^*∗*^	*r* = 0.011; *P* = 0.933	*r* < 0.001; *P* = 0.997
QUICKI	*r* = −0.633; *P* < 0.001^*∗*^	*r* = −0.555; *P* < 0.001^*∗*^	*r* = −0.056; *P* = 0.638
TC (mM)	*r* = 0.194; *P* = 0.027	*r* = 0.063; *P* = 0.640	*r* = 0.137; *P* = 0.249
TG (mM)^*∗*^	*r* = 0.337; *P* < 0.001^*∗*^	*r* = 0.396; *P* = 0.002^*∗*^	*r* = 0.171; *P* = 0.147
LDL-C (mM)	*r* = 0.159; *P* = 0.069	*r* = −0.223; *P* = 0.092	*r* = 0.077; *P* = 0.519
HDL-C (mM)	*r* = −0.596; *P* < 0.001^*∗*^	*r* = −0.578; *P* < 0.001^*∗*^	*r* = −0.426; *P* < 0.001^*∗*^
Uric acid (*μ*mol/L)	*r* = 0.172; *P* = 0.049	*r* = 0.493; *P* = 0.000^*∗*^	*r* = 0.067; *P* = 0.572
ALT (IU/L)^*∗*^	*r* = 0.157; *P* = 0.073	*r* = 0.223; *P* = 0.092	*r* = 0.183; *P* = 0.121
AST (IU/L)	*r* = 0.008; *P* = 0.923	*r* = 0.102; *P* = 0.445	*r* = 0.120; *P* = 0.313

BMI: body mass index; WHR: waist-to-hip ratio; FINS: fasting blood insulin; FBG: fasting blood glucose; HOMA-IR: homeostasis model assessment of insulin resistance; HOMA-%*β*: homeostasis model assessment of *β* cell function; TC: total cholesterol; TG: triglyceride; HDL-C: high-density lipoprotein cholesterol; LDL-C: low-density lipoprotein cholesterol; QUICKI: Quantitative Insulin Sensitivity Check Index; ALT: alanine aminotransferase; AST: aspartate aminotransferase; NAFLD: nonalcoholic fatty liver disease.

^*∗*^ *P* < 0.05.
